# Diet-Related Knowledge and Physical Activity in a Large Cohort of Insulin-Treated Type 2 Diabetes Patients: PROGENS ARENA Study

**DOI:** 10.1155/2016/2354956

**Published:** 2016-09-14

**Authors:** Tomasz Klupa, Michał Możdżan, Janina Kokoszka-Paszkot, Magdalena Kubik, Małgorzata Masierek, Margerita Czerwińska, Maciej T. Małecki

**Affiliations:** ^1^University Hospital, Krakow, Krakow, Poland; ^2^Department of Metabolic Diseases, Jagiellonian University, Krakow, Poland; ^3^Outpatient Diabetes Clinic, University Hospital in Lodz, Lodz, Poland; ^4^H. Klimontowicz Hospital, Gorlice, Poland; ^5^Outpatient Diet Clinic Fit & You, MedEvac Medical Center, Łódź, Poland; ^6^BIOTON S.A., Warszawa, Poland; ^7^Outpatient Diabetes Clinic, Polanica-Zdrój, Poland

## Abstract

There is no doubt that behavioral intervention is crucial for type 2 diabetes mellitus (T2DM) prevention and management. We aimed to estimate dietary habits and diet-oriented knowledge as well as the level of physical activity in 2500 insulin-treated Polish type 2 diabetes mellitus (T2DM) patients (55.4% women). The mean age of the study participants was 64.9 ± 9.3 years, mean BMI was 31.4 kg/m^2^ ± 4.5, mean diabetes duration was 12.4 ± 6.9 years, and mean baseline HbA1c was 8.5%  ± 1.2. At the study onset, all the patients completed a questionnaire concerning health-oriented behavior. Results showed a significant lack of diet-related knowledge. For example, only 37.5% recognized that buckwheat contains carbohydrates; the percentage of correct answers in questions about fruit drinks and pasta was 56.4% and 61.2%, respectively. As for the physical activity, only 57.4% of examined T2DM patients declared any form of deliberate physical activity. To conclude, the cohort of poorly controlled insulin-treated T2DM patients studied by us is characterized by insufficient diet-related knowledge and by a very low level of physical activity. Further studies on other populations of insulin-treated T2DM patients are required to confirm these findings.

## 1. Introduction

There is no doubt that behavioral intervention is crucial for type 2 diabetes mellitus (T2DM) prevention and management. As the prevention of the disease is concerned, it has been demonstrated that the incidence of type 2 diabetes could be reduced by almost 60% among patients with prediabetes through a weight loss resulting from changes in a diet and physical activity [1, 2]. Some data suggest that changes in patients' behavior may even lead to the remission of the recently diagnosed disease [3].

The behavioral intervention is also essential for the management of established diabetes. Nutritional therapy is recognized as one of the pillars of diabetes treatment [4–6]. Failure to achieve and maintain glycemic goals is often attributed to problems with implementing and following dietary recommendations [7]. The exercise has been shown to increase insulin sensitivity, control weight, and improve cardiovascular risk profiles [8–10]. Some studies based on limited populations suggest that the physical level is low among T2DM individuals [11, 12]; starting insulin therapy may be associated with an even further decrease in physical activity [13]. However, large scale data concerning physical activity in insulin-treated T2DM patients are missing.

## 2. The Aim of the Study

We aimed to estimate dietary habits and diet-oriented knowledge as well as the level of physical activity in a large cohort of insulin-treated type 2 diabetes mellitus (T2DM) patients. All were participants of the PROGENS ARENA study, which was designed to assess the role of behavior-oriented educational intervention in T2DM subjects.

## 3. Materials and Methods

There were 2500 insulin-treated Polish T2DM patients (55.4% women) included in the study. All of them were treated by specialists, diabetologists, and were recruited from 250 outpatient clinics distributed all over the country. 35% of them were country dwellers, 39.8% were small city dwellers (below 100 000 inhabitants), 13.8% were medium-sized city dwellers (from 100 000 to 500 000 inhabitants), and 11.4% were large city dwellers (above 500 000 inhabitants).

The doctors participating in the study were supposed to recruit first 10 consecutive patients seen on regular visits meeting following inclusion criteria:

- Diagnosis of T2DM based on clinical criteria
- Age above 18 years
- BMI < 40 kg/m^2^
- HbA1c > 7%
- Treatment with insulin for at least 12 months with noncompliance

Exclusion criteria included types of diabetes mellitus other than type 2, history of serious cardiovascular diseases (myocardial infarction or stroke within the last 3 months; heart failure, NYHA period IV; Angina Pectoris of III grade and IV grade, according to CCS; unstable hypertension > 180/100 mmHg, despite the use of antihypertensive drugs), impairment of renal function (GFR < 30 mL/min, creatinine > 135 *μ*mol/L), severe liver damage (tripling the AST and ALT levels above normal), with the exception of nonalcoholic fatty liver, modifications of dosage of medication which can affect the metabolism of corticosteroids (with the exception of the inhaled preparations), ACTH, and interferon, and chronic mental illness.

Additional exclusion criteria included addiction to alcohol and drugs, participation in clinical trials in the last 3 months, allergy to insulin or any of the components of the preparation, pregnancy or breast-feeding, and human insulin therapy (Gensulin M30, Gensulin M40, Gensulin M50, and Gensulin R) longer than 8 weeks.

All the patients filled a validated questionnaire concerning their physical activity and dietary habits/knowledge. Questions and answers to choose from the questionnaire are as follows:

(1) Did you set a specific goal of losing weight?
  (i) Yes, I want to lose weight by … kg
  (ii) No, I haven't done it yet
  (iii) No, I have no such need
(2) How many meals do you eat a day?
  (i) Number of main meals: …
  (ii) Number of smaller meals or snacks: …
(3) Please describe your eating habits:
  (i) I eat regular meals
  (ii) I follow a diet
  (iii) I used to sneak every now and then
  (iv) I do not eat regularly
  (v) I do not follow a diet
  (vi) I do not eat between meals
  (vii) Other variants. Which ones?
(4) Which of the following is the main meal for you?
  (i) Breakfast
  (ii) Elevenses
  (iii) Dinner
  (iv) Afternoon snack
  (v) Supper
  (vi) All the same
(5) Which of the following products contains carbohydrates?
  (i) Bread
  (ii) Yogurts
  (iii) Sausages
  (iv) Fruit drinks
  (v) Coca-Cola
  (vi) Potatoes
  (vii) Carrots
  (viii) Buckwheat groats
  (ix) Sweets
  (x) Pasta/noodles
(6) What percentage of carbohydrates should you consume per day?
  (i) <10%, better to avoid them
  (ii) 10–20%
  (iii) 21–30%
  (iv) 31–40%
  (v) 41–50%
  (vi) ≥51%
(7) Do you know what the glycaemic index of a product informs you about?
  (i) No, I do not know it exactly
  (ii) Yes, it informs me about how much a blood sugar level rises after eating this particular product
(8) Which of the following products have a low glycaemic index?
  (i) Raw carrots
  (ii) Wholemeal rye bread
  (iii) A wheat bread roll
  (iv) Overcooked wheat pasta
  (v) Kohlrabi
  (vi) Bananas
(9) Does the amount of meat you eat result in a large increase in blood glucose?
  (i) Yes
  (ii) No
  (iii) I do not know
(10) What types of meat do you choose the most?
  (i) Pork
  (ii) Chicken
  (iii) Turkey
  (iv) Beef
  (v) Game
  (vi) I do not eat meat
  (vii) Fish
(11) What kind of fat do you use for cooking or frying?
  (i) Lard, pork fat
  (ii) Olive oil
  (iii) Butter
  (iv) Margarine
  (v) Rapeseed oil
  (vi) Sunflower oil
  (vii) Other oils. Which ones?
(12) Do fruit yogurts contain sugar in their composition?
  (i) Yes
  (ii) No
  (iii) I do not know
(13) Is canned fruit just as healthy as fresh?
  (i) Yes
  (ii) No, it is additionally sweetened
(14) In what order are ingredients listed on a product label?
  (i) The decision depends on a manufacturer
  (ii) Always starting from the component which is the most to that one which is the least
(15) Is a drink the same as juice?
  (i) Yes, both are derived from fruit
  (ii) No, drinks are sweetened with sugar or glucose-fructose syrup
(16) What is the healthiest potion to quench your thirst?
  (i) Water
  (ii) Fruit drinks/juices
  (iii) Tea
  (iv) Other beverages. Which ones?
(17) Does the amount of time for a meal matter?
  (i) Yes, it is better to east slowly
  (ii) No, it does not matter
(18) Are you physically active?
  (i) Yes
  (ii) No
(19) What form of exercise you take?
  (i) Of low intensity (e.g., walking, seasonal work in the garden, Nordic walking)
  (ii) Of moderate intensity (e.g., swimming, running, cycling)
  (iii) Of high intensity (e.g., professional/competitive sport)
(20) How much time per week do you spend on physical activity?
  (i) Not at all
  (ii) Once-twice a week
  (iii) Three-four times a week
  (iv) Five-six times a week
  (v) Every day

The questionnaire had been originally prepared for the PROGENS study in its major part; however present research tool incorporates, validated, and universally used Diabetes Treatment Satisfaction Questionnaires (DTSQC). Moreover, the PROGENS questionnaire, as a whole, underwent the phase of trial tests (i.e., pilot study), which allowed the authors to verify its contents.

As for statistical analysis, numerical data were described as their average ± standard deviation values; categorical outcomes were presented as percentages. In order to estimate the significance of differences, the Chi-squared test of independence and tests for trend and log-linear models were carried out. A level of *p* < 0.05 was considered statistically significant. All the statistical procedures were carried out by the use of Stata/Special Edition, release 12.1 (College Station, Texas, USA).

The study design was accepted by Bioethical Committee of the Jagiellonian University; all patients gave a written informed consent.

## 4. Results

The mean age of the study participants was 64.9 ± 9.3 years, mean BMI was 31.4 kg/m^2^  ± 4.5, mean diabetes duration was 12,4 ± 6.9 years, and mean baseline HbA1c was 8.5%  ± 1.2. The proportion of subjects using prandial short acting human insulin was 50.3% (in the model of multiple daily injections or as a component of a premixed regimen; patients on basal insulin only were not included in the study); others used rapid acting insulin analogues. At the study onset, all the patients completed a questionnaire concerning health-oriented behavior, including dietary habits, diet-related knowledge, and physical activity.

There were 55.9% of the study group declaring to follow the dietary rules recommended in diabetes. The majority of examined T2DM patients (68.7%) reported understanding the meaning of the term “Glycemic Index.” As many as 86.6% individuals indicated that they understood the importance of slow food consumption. There were no significant differences in answers distribution with respect to patients' origin.

**Figure 1:**
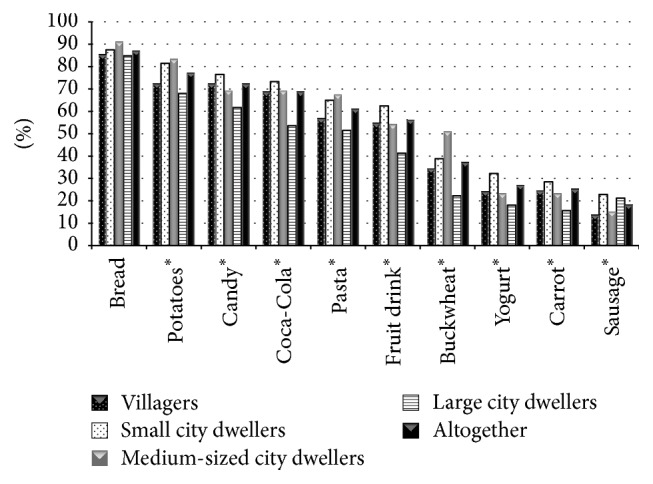
Products recognized as ones containing carbohydrates by study participants (an asterisk near the category name indicates a statistically significant difference by domicile; *p*< 0,005).

However, the answers to more specific questions showed a significant lack of diet-related knowledge. A relatively small percentage of responders recognized that commonly consumed food or drinks contain carbohydrates. Surprisingly, the percentage of correct answers was the smallest among large city dwellers (Figure 1).

For example, only 37.5% recognized that buckwheat contains carbohydrates; the percentage of correct answers in questions about fruit drinks and pasta was 56.4% and 61.2%, respectively. For large city dwellers, the percentage of correct answers was 22.7%, 41.7%, and 51.5% (*p* < 0.05). Additionally, 27.4% and 22.8% of T2DM patients (for large city dwellers: 37.9% and 31.8%, *p* < 0.05) answered that candy and potatoes, respectively, did not contain carbohydrates.

**Figure 2:**
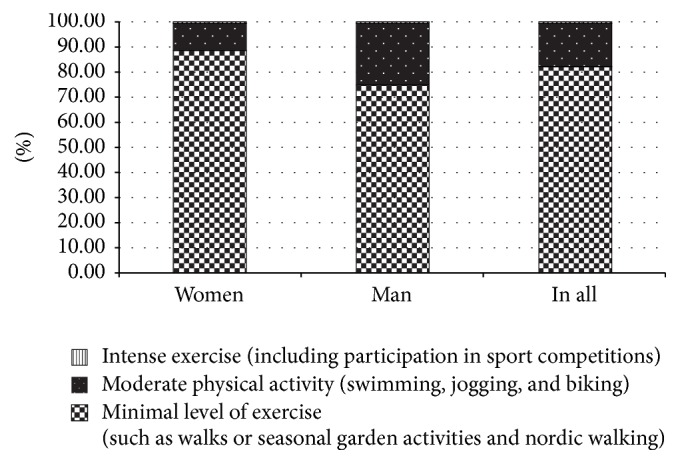
The level of physical activity declared by study participants (the “Intense exercise (…)” category not visible due to very small values).

As far as physical activity is concerned, only 57.4% of examined T2DM patients (51.8% women and 64.4% men) declared any form of deliberate, leisure time physical activity. Most of the group (82.3%) reported a minimal level of exercise, such as walks or seasonal garden activities. Only 17.4% of patients declared moderate physical activity (swimming, jogging, and biking); it is of note that this level of activity was declared more often by men than women (24.9% versus 11.4%). Intense exercise (including participation in sport competitions) was reported only by 0.3% of the study participants (Figure 2). There were no statistically significant differences with respect to the origin of patients.

**Figure 3:**
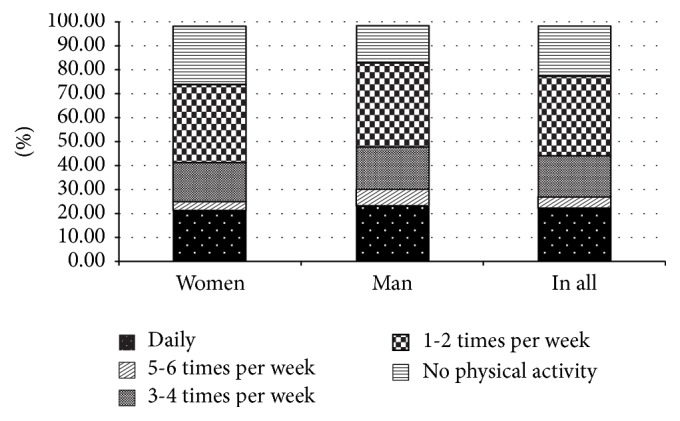
Frequency of any form of physical activity declared by study participants.

The majority of T2DM patients declared undertaking any form of deliberate physical activity only 1-2 times per week. (Figure 3). Exercise was performed on a daily basis only by 22.3% of T2DM patients but only by 7.6% out of surveyed large city dwellers; *p* < 0.05.

Interestingly, despite the high average BMI of the study population, only 46.4% of patients declared a desire to lose weight. For large city dwellers, numbers were even smaller (36.4%, *p* < 0.05).

## 5. Discussion

Here we present questionnaire-based data concerning the diet-related awareness, dietary habits, and physical activity in large cohort of Polish insulin-treated patients. To our knowledge, it is one of the largest sets of data concerning these issues in Poland.

As the diet is concerned, our study indicated that there is a strong discrepancy between the theoretical, self-declared, and actual tested levels of diet-related knowledge in insulin-treated T2DM patients. The majority of questions in the detailed part of the questionnaire were related to carbohydrates, the most potent modifier of glycemic patterns in patients with T2DM [4]. Unfortunately, the study showed a very poor level of practical knowledge concerning carbs.

Thus, one could expect that the failure to achieve good glycemic control in the study group (baseline average HbA1c, 8.5%) to large degree could be attributed to the discrepancies between insulin dosing and carbohydrate consumption. We postulate that carbohydrate-oriented practical dietary education should be a priority for insulin-treated T2DM patients.

To our surprise, large city dwellers were characterized by the poorest carbohydrate knowledge and the lowest desire to lose weight. That could reflect a higher engagement in diabetes management among the country or small/medium-sized city dwellers or simply reflect recruitment bias.

As for the physical activity of patients with type 2 diabetes, the results of our study support recently released data; however, our study was limited to insulin-treated patients only, which was not the case in other papers. Avramopoulos et al. showed that in Greek population 79.7% of the patients exerted none or light physical activity [14]; however, only less than quarter of studied group was treated with insulin. Linmans et al. also reported that a large part of the T2DM patients reported having a deficient physical activity level (35% according to patients, 47% according to healthcare professionals) [15]; however, again, minority of them only were insulin-treated. Preiss et al. indicated further decrease in physical activity in individuals relatively inactive at baseline after the diagnosis of type 2 diabetes [16]. This means that extremely low level of physical activity among T2DM patients is a universal problem and should be one of the most important educational and therapeutical targets. This phenomenon together with a deficiency of knowledge concerning diet could have contributed to the failure to achieve glycemic targets in our cohort.

Our study has some obvious limitations. First of all, it was based on questionnaires; thus answers, especially those to general questions, could be biased by multiple factors. One may speculate, for instance, that sex differences in a declared level of physical activity could be related to difference in being ready to admit a low physical activity between man and women rather than actual frequency of exercise. It was shown that T2DM patients tend to overestimate their physical activity and there may be sex differences in this field [12, 15]. Additionally, it should be underlined that due to the nature of recruitment protocol the study population may not be fully representative for the participating centers. Furthermore, the distribution of the centers was not even throughout the country; thus the results may be affected by bias of some degree related to this fact.

Another limitation of the study may be due to wide exclusion criteria that were used by us. One of the reasons to incorporate such wide exclusion criteria was to avoid patients who were supposed to modify their diet or type/level of physical activity due to the presence of late complications of diabetes and/or concomitant diseases on the top of behavioral intervention prescribed for majority of patients with diabetes.

Despite some limitations, we believe that our data are of high clinical importance. On one hand, they show that insulin-treated T2DM patients in Poland are far from meeting clinical recommendations for physical activity [17, 18]. On the other hand, they indicate the need for more practical dietary education.

## 6. Conclusions

In conclusion, there is a strong discrepancy between the theoretical, self-declared, and actual tested levels of diet-related knowledge in insulin-treated T2DM patients. A particularly surprising finding is the low level of practical knowledge concerning carbohydrates, especially among large city dwellers. Insulin-treated T2DM patients are characterized by a very low level of physical activity.
